# Effects of visual impairment on mobility functions in elderly: Results of Fujiwara-kyo Eye Study

**DOI:** 10.1371/journal.pone.0244997

**Published:** 2021-01-29

**Authors:** Kimie Miyata, Tadanobu Yoshikawa, Akihiro Harano, Tetsuo Ueda, Nahoko Ogata

**Affiliations:** 1 Department of Ophthalmology, Nara Medical University, Kashihara, Nara, Japan; 2 Department of Orthopedics, Yamatotakada Municipal Hospital, Yamatotakada, Japan; University of Ulster, UNITED KINGDOM

## Abstract

The aim of this study was to determine whether there is a significant association between a visual impairment (VI) and mobility functions in an elderly Japanese cohort. The subjects of this study were part of the Fujiwara-kyo Eye Study, a cross sectional epidemiological study of elderly individuals conducted by Nara Medical University. Participants were ≥70-years who lived in the Nara Prefecture. All underwent comprehensive ophthalmological examinations, and a VI was defined as a best-corrected visual acuity (BCVA) worse than 20/40 in the better eye. The associations between the BCVA and walking speed and one-leg standing time were determined. The medical history and health conditions were evaluated by a self-administered questionnaire. A total of the 2,809 subjects whose mean age was 76.3 ± 4.8 years (± standard deviation) were studied. The individuals with a VI (2.1%) had significantly slower walking speeds and shorter one-leg standing times than that of the non-VI individuals (1.5±0.4 vs 1.7±0.4 m/sec, *P*<0.01; 17.1±19.6 vs 27.6±21.3 sec, *P*<0.01, respectively). Univariate logistic regression found that the odds ratio (OR) for the slower walking speed (<1 m/sec) in the VI individuals was significantly higher at 7.40 (3.36–16.30;95% CI, *P* <0.001) than in non-VI individuals. It was still significantly higher at 4.50 (1.87–10.85;95% CI, *P* = 0.001) in the multivariate logistic regression model after adjusting for the BCVA, age, sex, current smoking habit, and health conditions. Our results indicate that the walking speed and one-leg standing times were significantly associated with VI.

## Introduction

It has been reported that the risk of the risk of mobility difficulties increase after 65-years-of-age [1], and mobility difficulties may lead to higher risks for more disabilities, difficulties in activities of daily living (ADLs), institutionalization, and survival [2,3]. Chronic health diseases are the most common causes for physical disabilities that arise at older ages [3, 4], and visual impairments (VIs) have also been considered to be associated with the functional difficulties [4].

In earlier studies, we found that VIs affected the cognitive function of the elderly subjects [5,6]. Our next question was whether the VIs would also affect the physical functions of older individuals. Earlier studies examined the effect of VI on physical mobility, and they reported that individuals with VIs had slower walking speeds, had more falls, and had more mobility difficulties than non-visually impaired individuals [7–11]. Thus, VI is not only a sensory disability but a significant deterrent to mobility functions. However, many these previous studies identified mobility difficulty by self-reporting [7–11].

The Salisbury Eye Evaluation Studies reported that individuals with VIs had greater mobility disabilities than the non-VIs individuals [12,13]. However, how VI effects on mobility has not been fully examined.

The walking speed is a strong predictor of disability and death in older adults [14–17], and is used as a criterion for sarcopenia and frailty in the evaluation of health and mobility functions in elderly populations [18–21]. A shorter one-leg standing time is believed to indicate a weakness of the leg muscles which then enhances the risk of falling and locomotor disabilities [22,23].

The purpose of this study was to determine the effect of VIs on the physical capabilities. To accomplish this, we measured the walking speed and the one-leg balancing times in an elderly population whose VIs were also measured.

## Subjects and methods

### The Fujiwara-kyo Study

A detailed description of the Fujiwara-kyo Study has been published [24,25]. Briefly, this was a cohort study whose purpose was to identify factors related to the maintenance of a healthy life, prevention of physical weakness, and improve the functional capacities and quality of life (QOL) of an elderly population in Japan [24,25]. The subjects consisted of residents in the Nara Prefecture who were ≥65 years and living independently in their homes. The first examinations were performed in 2007, and the second survey was conducted in 2012. The examinations included a basic interview to obtain socio-demographic data, overall medical conditions, and histories of medical treatments. Participants brought filled-in self-administered questionnaires which had been mailed to their homes in advance of the live examination. The answers of all questions to the participants were checked by a nurse in a face-to-face during the live interviews, and the missing items were collected and written answers were confirmed.

### The Fujiwara-kyo Eye Study

An ophthalmological survey, the Fujiwara-kyo Eye study, was conducted for the first time as part of the Fujiwara-kyo Study at the second survey during February to November 2012. The data presented in this manuscript were collected from the examinations done in 2012 [5,6]. The subjects recruited at the initial survey in 2007 were ≥65-years-old and were 5-years older in 2012.

The surveys were conducted in accordance with the tenets of the Declaration of Helsinki, and the protocol was approved by the Ethics Review Board of Nara Medical University. A signed informed consent form was obtained from all participants.

#### Ophthalmological examinations

*Visual acuity*. The refractive error (spherical equivalent) was determined by an auto refractometer and keratometer (ARK-700A, Nidek, Aichi, Japan). The data of the refractive errors were used as useful references to obtain the best-corrected visual acuity (BCVA). The uncorrected and the BCVA of both eyes were measured with a Landolt ring chart at 5 m by well-trained orthoptists. To obtain the BCVA, orthoptists used corrective lenses placed in trial frames for each participant. A VI was defined as BCVA worse than 20/40 in the better-seeing eye for the statistical analyses. This cut-off BCVA corresponded to the American Association of Ophthalmology categorization of a visual impairment which was defined as the distance BCVA worse than 20/40 in the better-seeing eye [26]. The decimal BCVA was converted to the logarithm of the minimum angle of resolution (logMAR) units for the statistical analyses.

### Walking speed test

The walking speed was assessed over a distance of 10 m after 2 m for acceleration. Participants were required to walk as fast as possible at their maximal speed. Participants walked on a flat floor wearing walking shoes or sneakers. An attendant followed each participant during the speed walking test and one-leg standing test to protect the participants from an unexpected occurrences and avoid injuries from falls. In all tests, no accidental falls happened, and all participants finished the mobility tests safely. The walking time in 0.1 sec periods was measured with an automatic measurement system (10 m Walking Speed Measurement Model T.K.K.5801 and 5807; Takei Scientific Instruments, Niigata, Japan), and the walking speed (m/s) was calculated by dividing distance (10 m) by the time. The mean results of two trials was used for the statistical analysis. We defined a slow walking speed as <1 m/sec according to the sarcopenia criteria of Japanese version of the CHS (J-CHS criteria) [18], the International Waterlily & Water Gardening Society (IWGS) [20], and the new criteria of Asian Working Group for Sarcopenia (AWGS) of 2019 [21]. We also analyzed a slow walking speed of ≤0.8 m/sec according to the European Working Group on Sarcopenia in Older People (EWGSOP) criteria for sarcopenia [19].

### One-leg standing time

Participants were instructed to stand on their preferred leg for as long as possible with their eyes opened and hands on their waist. The duration of the one-leg stand was until balance was lost. The one-leg standing time represents the static balance of the body, and a shorter one-leg standing time suggests an increased risk of falling [22,23]. If the participant exceeded 60 s, the time was recorded as 60 s. The mean of two trials was used for the statistical analyses.

### General information and health conditions

The medical history and medication information were obtained by a self-administered questionnaire. The presence of systemic hypertension was based on a self-reported diagnosis and current antihypertensive therapy. The presence of diabetes mellitus was based on a self-reported diagnosis, current anti-diabetic therapy, and fasting plasma glucose and glycated hemoglobin levels. The presence of hip arthritis, knee arthritis, leg pain, ankle and foot pain, low back and buttock pain, and sciatica were defined base on self-administered questionnaire and self-reported previous diagnosis.

We also determined the presence of covariates; body mass index (BMI), smoking status, and a number of comorbid conditions. BMI was calculated as body weight (kg) divided by height (m) squared, and it was categorized into three groups: low (<18.5 kg/m^2^), normal (18.5 to <25 kg/m^2^), and high (≥25 kg/m^2^).

### Statistical analyses

The significance of the differences of the findings in the VI and non-VI participants was determined by unpaired *t* tests and chi-square tests. The significance of the association between walking speed or one-leg standing time and other parameters was determined by linear regression analyses. The multivariate analyses were adjusted for age, sex, BMI, current smoking, and the number of other health conditions (Table 1). The odds ratios (OR) and 95% confidence intervals (CI) for slow walking speed (<1 m/sec and ≤0.8 m/sec), the visual impairments, age, sex, BMI, current smoking, and the health conditions were calculated by logistic regression models. The results of earlier research have indicated that the risk of both VIs and disability increases nonlinearly with age [7]. To take this nonlinear association into consideration, the age at baseline was grouped into 5 years periods, e.g., 65 to 69, 70 to 74, 75 to 79, and over 80 years. The statistical analyses were performed with the SPSS (version 22.0; SPSS Inc., Chicago, IL). A *P* <0.05 was taken to be significant.

**Table 1:** Basic and clinical characteristics of 2809 participants.

Characteristics	All	VI	Non-VI	*P*
Number (%)	2809	60 (2.1)	2749	
Age, mean ± SD, years	76.3 ± 4.8	79.2 ± 6.2	76.2 ± 4.8	<0.01
Sex (men), number (%)	1482 (52.8)	26 (43.3)	1456 (53.0)	0.13
BCVA, mean ± SD, logMAR units	-0.02 ± 0.14	0.53 ± 0.18	-0.03 ± 0.11	<0.01
Walking speed, mean ± SD, m/seconds	1.7 ± 0.4	1.5 ± 0.4	1.7 ± 0.4	<0.01
Average one-leg standing time with eyes opened, mean ± SD, seconds	27.4 ± 21.3	17.1 ± 19.6	27.6 ± 21.3	<0.01
BMI, mean ± SD, kg/m^2^	22.7 ± 2.9	22.5 ± 3.3	22.7 ± 2.9	0.62
Current smoking, number (%)	1171 (42.3)	21 (36.2)	1150 (42.4)	0.34
Number of other health conditions, number (%)				
0	503 (17.9)	12 (20.0)	491 (17.9)	
1	623 (22.2)	12 (20.0)	611 (22.2)	
2	616 (21.9)	9 (15.0)	607 (22.1)	
3	657 (23.4)	17 (28.3)	640 (23.3)	
over 4	410 (14.6)	10 (16.7)	400 (14.6)	0.66
Health conditions				
Hip arthritis, number (%)	45 (1.6)	0 (0.0)	45 (1.7)	0.31
Knee arthritis, number (%)	580 (20.7)	11 (18.3)	569 (20.7)	0.65
Leg pain, number (%)	1328 (47.3)	29 (48.3)	1299 (47.3)	0.87
Ankle and foot pain, number (%)	132 (4.7)	4 (6.7)	128 (4.7)	0.47
Low back and buttock pain, number (%)	553 (19.7)	16 (26.7)	537 (19.5)	0.17
Sciatica, number (%)	99 (3.5)	2 (3.3)	97 (3.5)	0.94
arrhythmia, number (%)	232 (8.3)	3 (5.0)	229 (8.3)	0.35
Ischemic heart disease, number (%)	77 (2.7)	1 (1.7)	76 (2.8)	0.61
Diabetes, number (%)	414 (14.7)	10 (16.7)	404 (14.7)	0.67
Hypertension, number (%)	1435 (51.8)	30 (51.7)	1405 (51.8)	0.99
COPD, number (%)	129 (4.7)	1 (1.7)	128 (4.7)	0.28
Stroking, number (%)	172 (6.1)	6 (10.0)	166 (6.0)	0.21
Cancer, number (%)	420 (15.0)	14 (23.3)	406 (14.8)	0.07

SD, standard deviation; BMI, Body mass index; BCVA, the best-corrected visual acuity; COPD, chronic obstructive pulmonary disease.

## Results

### Demographics of participants

A total of 2,809 subjects whose mean age was 76.3 ± 4.8 years (mean ± standard deviation, SD) were studied. There were 1,482 men and 1327 women, and 60 (2.1%) of the participants were categorized as VI, and 2,749 (97.9%) were categorized as non-VI. The VI participants were significantly older but there was no significant difference in the sex distribution, BMI, smoking habit, and general health conditions between the two groups (Table 1).

### Visual impairment status and mobility performance

The participants in the VI group had significantly slower walking speeds of 1.5 ± 0.4 m/sec than did the non-VI group at 1.7 ± 0.4 m/sec (*P* <0.05; Table 1). In addition, the individuals with VI had a significantly shorter one-leg standing time of 17.1 ± 19.6 sec than that of the non-VI group at 27.6 ± 21.3 sec (*P* <0.05; Table 1).

### Associations between walking speed or one-leg standing time and other demographic parameters

The visual acuity, age, sex, current smoking habit, and the number of other health conditions were significantly associated with the walking speed and one-leg standing time (both *P* <0.001) as determined by linear regression analyses of univariate models. In the multivariate analyses, the visual acuity, age, sex, and the number of other health conditions were significantly associated with the walking speed and one-leg standing time *(P* <0.001; Tables 2 and 3). The BMI was significantly associated with one-leg standing time but not significantly associated with the walking speed.

**Table 2:** The associations between walking speed and parameters.

	Univariate model		Multivariate model	
	Β	*P* value	Β	*P* value
BCVA	-0.554	<0.01	-0.266	<0.01
Age	-0.029	<0.01	-0.028	<0.01
Sex (male)	-0.199	<0.01	-0.223	<0.01
BMI	-0.001	0.786	-0.006	0.008
Current smoking	0.137	<0.01	-0.021	0.22
Number of other health conditions	-0.051	<0.01	-0.036	<0.01

BCVA, the best corrected visual acuity; BMI, Body mass index.

Multivariate model; Adjusted for age, sex, BMI, current smoking, and number of other health conditions.

**Table 3:** The associations between average one-leg standing time and parameters.

	Univariate model		Multivariate model	
	Β	*P* value	β	*P* value
BCVA	-32.09	<0.01	-17.11	<0.01
Age	-1.78	<0.01	-1.71	<0.01
Sex (male)	-4.68	<0.01	-6.09	<0.01
BMI	-0.71	<0.01	-0.98	<0.01
Current smoking	3.19	<0.01	-0.65	0.54
Number of other health conditions	-2.61	<0.01	-1.44	<0.01

BCVA, the best corrected visual acuity; BMI, Body mass index.

Multivariate model; Adjusted for age, gender, BMI, current smoking, and number of other health conditions.

### Odds ratio for slow walking speed (<1 m/sec and ≤0.8 m/sec) in participants with and without visual impairment

We set the cut-off for slow walking speed at 1.0 meter/sec [18,20,21] or ≤0.8 m/sec [19]. In the univariate logistic regression model, the OR for slow walking speed in the VI group was significantly higher (OR;7.40 at <1.0 m/sec, 95% CI; 3.36–16.30, *P* <0.001; OR;6.49 at ≤0.8 m/sec, 95% CI; 2.46–17.11, *P* <0.001, respectively) than for the non-VI group (Table 4).

**Table 4:** Odds ratio for slow walking speed in participants with and without visual impairment.

	Participants	
	with visual impairment (n = 60)	without visual impairment (n = 2749)
Slow walking speed (<1m/sec)		
Unadjusted OR (95% CI)	7.40 (3.36, 16.30)	1.0 (ref)
*P* value	<0.001	
Adjusted OR (95% CI)	4.50 (1.87, 10.85)	1.0 (ref)
*P* value	0.001	
Slow walking speed (≤0.8m/sec)		
Unadjusted OR (95% CI)	6.49 (2.46, 17.11)	1.0 (ref)
*P* value	<0.001	
Adjusted OR (95% CI)	3.51 (1.21, 10.15)	1.0 (ref)
*P* value	0.021	

BCVA, best corrected visual acuity; OR, Odd ratio; CI, confidence intervals.

Adjusted OR is adjusted for variables (age, sex, BMI, smoking, and health conditions). BMI was categorized 3 by underweight (<18.5 kg/m^2^), normal weight (18.5–24.9 kg/m^2^), and overweight or obese (≥25.0 kg/m^2^).

In the multivariate logistic regression model, adjustments were made for the BCVA, age, sex, current smoking habit, and the health conditions which were significant parameters in the univariate models. The OR for slow walking speed in the VI group was significantly higher (OR; 4.50 at <1.0 m/sec, 95% CI; 1.87–0.85, *P* = 0.001, OR;3.51 at ≤0.8 m/sec, 95% CI; 1.21–10.15, *P* = 0.021, respectively) than for non-VI group (Table 4). These data indicate that there is a specific association between VA and walking speed.

## Discussion

The population of older individuals has been rapidly increasing in advanced countries which have increased the cost of medical care and social security burdens for the government. To try to reduce or minimize the medical costs, governments must determine the factors associated with the increased medical care. One factor we have been concentrating on is the role played by the visual functions on the quality of life of older individuals.

The walking speed and standing one leg balance has been proposed to be strong predictors of disabilities and death of older adults [2,14–17,22,23,27,28].

Earlier studies suggested that older adults with VIs were more likely to report mobility difficulties and have slower walking speeds than their non-VIs individuals [7–11,29,30]. However, many of these studies identified the mobility difficulties by self-reporting [7–11]. For our study, we measured the walking speed and one-leg standing time, and our results clearly demonstrated that individuals with VIs had significantly slower walking speeds and significantly shorter one-leg standing times than did the non-VI individuals.

An earlier cohort study of Japanese rural individuals ages >65 years reported that the average walking speed over 10 meters was 1.33 meters/sec [16]. Our participants demonstrated relatively faster walking speed than that cohort, but the participants with VIs had a significantly slower walking speed than non-VIs participants. We suggest that the individuals with VI have a greater fear of falling than do the non-VI individuals. Thus, older adults with VI walk more slowly to avoid falling. Ayaki et al. reported a significant correlation between the walking speed and visual acuity, and they confirmed that a healthy binocular function is important for normal gait [31]. They compared the pre- and postoperative walking speed in cataract patients and reported a significant improvement in the walking speed after cataract surgery [31]. It is possible that the slowing of the walking speed is an adaptation at the onset of VIs, and that individuals with VIs walk slowly to maintain or improve their mobility safety.

The Salisbury Eye Evaluation Study reported that the interaction between age and VI status was not significant which indicated that the mobility speeds decreased at a similar rate in individuals with VIs and non-Vis [32]. However, the results of our study demonstrated that the unadjusted OR for slow walking speed of <1 m/sec was significantly high at 7.40 in individuals with VI, and after adjustments for the age and health conditions, the OR was still significantly high at 4.50. These results indicate that the VI status affected the walking speed more likely than age.

A shorter one-leg standing time indicates low overall body balancing ability which is a greater risk for falling. When the walking speed slows, it reduces the daily walking activity which then leads to a weakening of the leg muscles and reduces static balance of the body. Thus, shorter one-leg standing times indicate a weakness of the leg muscles which would increase the risk of falling and enhance locomotor disability [22,23].

Falls and fall-related fractures are a major public health problem among the older adults [33]. It has been reported that 30% of community-dwelling adults older than 65 years fall at least once annually with 5% of falls resulting in fractures and 10% resulting in other serious injuries [34]. We demonstrated that the walking speed and one-leg standing time were significantly associated with the BCVA. Further studies are warranted to confirm the effects of visual improvements on the walking speeds and one leg standing times.

There are some limitations in this study. This was a cross-sectional study which precludes assessments of causality, and this was a survey study in which extensive examinations of mobilities were limited.

In conclusion, our results demonstrate the negative effects of VIs on the mobility of older individuals. Thus, it is important for older individuals to have comprehensive ophthalmological examinations, and have all visual deficits corrected as best as they can.

## Supporting information

S1 Dataset (XLSX)
